# Cleidocranial Dysplasia Presenting With Mixed Dentition in a 28-Year-Old Male: A Case Report

**DOI:** 10.7759/cureus.65273

**Published:** 2024-07-24

**Authors:** Monitha Gollapudi, Swapnil Mohod, Nisarga R Mahajan, Aakanksha V Tiwari

**Affiliations:** 1 Department of Periodontology, Sharad Pawar Dental College and Hospital, Datta Meghe Institute of Higher Education and Research, Wardha, IND; 2 Department of Oral Medicine and Radiology, Sharad Pawar Dental College and Hospital, Datta Meghe Institute of Higher Education and Research, Wardha, IND; 3 Department of Oral Pathology, Sharad Pawar Dental College and Hospital, Datta Meghe Institute of Higher Education and Research, Wardha, IND

**Keywords:** ossification, permanent teeth, hypoplastic clavicles, cranial sutures, cleidocranial dysplasia

## Abstract

Cleidocranial dysplasia (CCD) is an inherited development anomaly of the skeletal system that is also classified as an autosomal dominant genetic disorder. This is due to a gene mutation on chromosome 6p21 that encodes core binding factor activity a-1 (CBFA1), a member of runt-related transcription factor 2 (RUNX2) found on the short arm of chromosome 6. CCD is a scarce condition and its occurrence is about one per million births. It primarily affects bones that are derived from both endochondral and intramembranous ossification. It is identified by certain clinical and radiological features including open cranial sutures and open anterior fontanelle, aplastic or hypoplastic clavicles, wormian bones, short stature, deformities of the pelvic bones, and various skeletal changes. Patients usually show class III malocclusion because of mandibular hyperplasia and mid-face hypoplasia. Vertical facial growth is reduced due to hypoplasia of the alveolar bone, and permanent teeth eruptions are failed. We reported a case of CCD in a 28-year-old who was referred to OPD for poor esthetics.

## Introduction

Cleidocranial dysplasia (CCD) is a genetic disorder of bones that originates from the mutation in the runt-related transcription factor 2 (RUNX2) and follows an autosomal dominant highly polymorphic skeletal trait with variable penalties that primarily involve the bones that are formed through the process of intramembranous ossification [1]. CCD has been documented a long time ago, with the earliest recorded reports being as early as 400 years ago. However, Yamamoto et al. in the year 1989 narrated a white male patient aged 28 years with 63 supernumerary teeth [2]. This condition affects 0.5-1/100,000 live births with equal prevalence in both males and females. These typically affect people between the ages of 10 to 15 years and exhibit abnormalities of dental and skeletal. However, 40% of cases occur without the indication of an inherited gene [3]. 

CCD patients are of short stature, slender with long necks, and drooped shoulders. The most distinct feature of CCD patients is the partial or complete absence of a clavicle which results in the patient approximating the shoulders in front of the chest. The absence of clavicle occurs only in 10% of cases [4]. They also present with late closure of fontanels and open skull sutures. The base of the skull is reduced in growth which therefore results in brachycephalic head and hypertelorism [5]. Frontal bossing may also be present. Scoliosis is also seen in many cases. The mental condition of these individuals is usually normal [6]. Other systemic problems include recurrent ear and sinus infections, upper respiratory complications, minor motor delay shown in children under 5 years of age, and a high incidence of cesarean section. Radiographic examination is a critical confirmatory diagnostic tool.

There are countless variants of dental abnormalities including the delayed shedding of deciduous teeth, the unerupted secondary or permanent dentition, underdevelopment maxilla with high arched palate, poorly outlined paranasal sinuses, multiple supernumerary teeth that imitate a lot like premolars, severe malocclusion and cross-bites and delayed maturation of permanent teeth [1]. CCD patients in the adult group display mixed dentition. It was observed that the affected individuals have a fatty formation around the affected teeth. Teeth abnormalities include cementum and enamel hypoplasia, microdontia, and root dilacerations [7]. Some studies have shown that the failure of eruption of teeth is due to an increase in the amount of acellular cementum and the absence of cellular cementum in the root of the affected roots [8].

This results in delayed ossification of the bone precursors mainly at junctions leading to either defective ossification or absence of ossification of parts of skeletal structures [9]. Thus, CCD is initiated through heterozygotic mutations in human beings. The gene responsible for the pathogenesis of CCD is located at 6p21, more specifically Core Binding Factor a-1 (Cbfa1). Cbfa1 (RUNX2) is involved in the process of growth and development of the skeleton in humans by regulating the process of endochondral and intramembranous ossification. It also controls the gene expression of dental epithelial-mesenchymal cells and the deficit of Cbfa1 can also lead to dental abnormalities [10]. This is a case report of a 28-year-old male patient diagnosed with CCD.

## Case presentation

**Figure 1:**
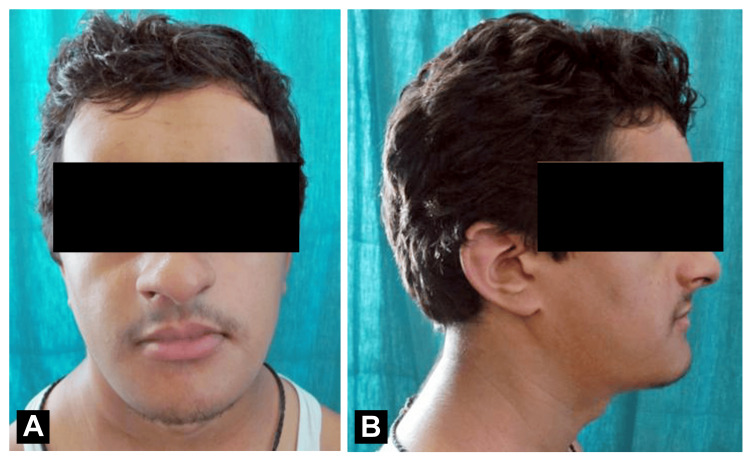
(A) Frontal view showing frontal bossing, wide nasal bridge, and hypertelorism; (B) Lateral view showing increased head circumference.

A 28-year-old male patient reported to the Department of Oral Medicine and Radiology at Sharad Pawar Dental College and Hospital, Datta Meghe Institute of Higher Education and Research (DMIHER), Wardha with a chief complaint of poor esthetics. The patient was moderately nourished. The patient has short stature with stunted growth of hands and legs. Past medical and family history are not significant. Extraoral examination revealed frontal bossing, sunken eye, hypertelorism, increased head circumference, and wide nasal bridge (Figure 1A, 1B).

**Figure 2:**
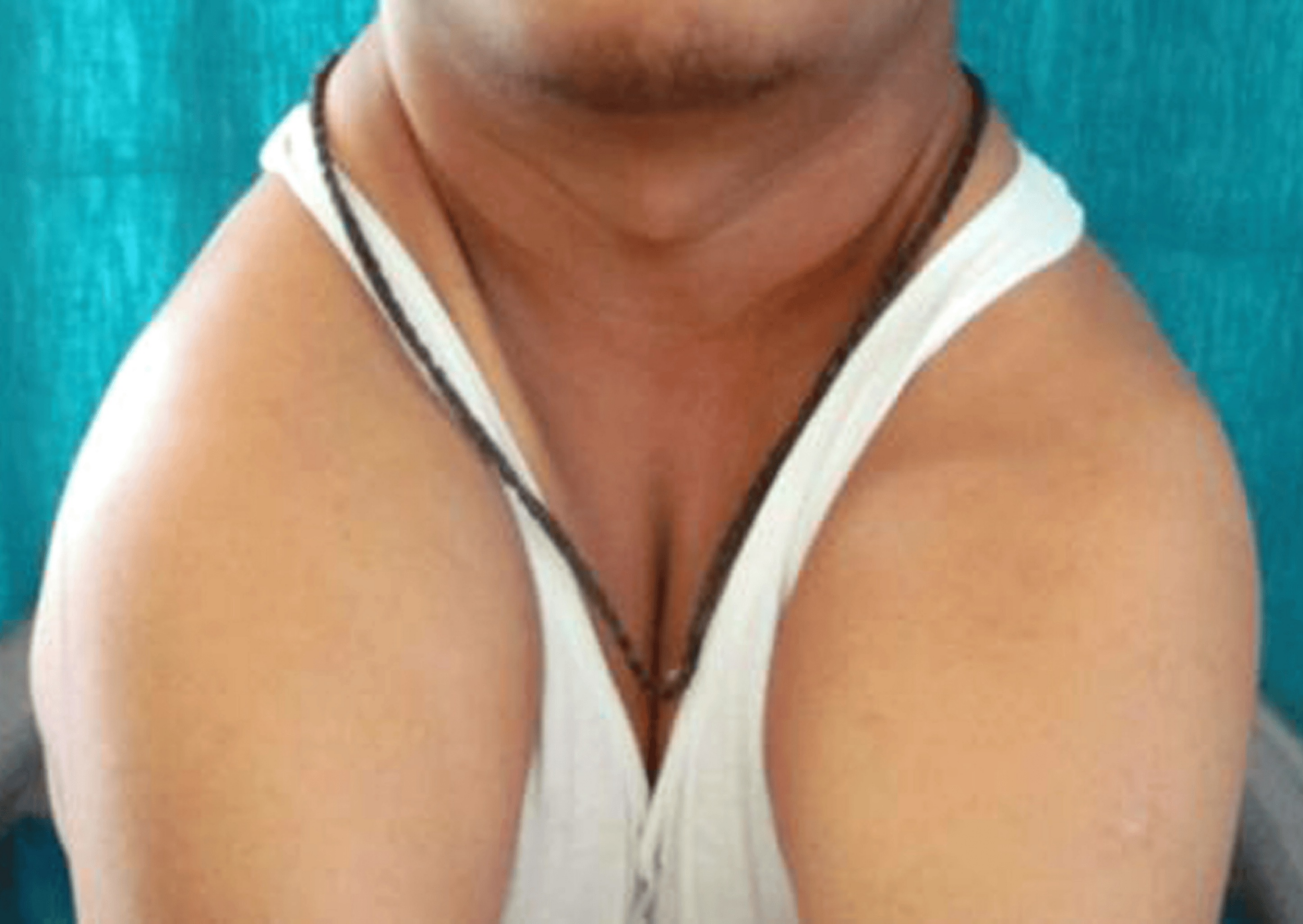
Showing hypermobility of shoulders.

The patient exhibits an oval facial form and lips are competent. There was hypermobility of the shoulders and both could be completely approximated in the midline due complete absence of clavicles when asked to do so (Figure 2).

Intraoral examination revealed that the patient has mixed dentition. Table 1 shows the teeth present in the oral cavity according to the FDI (Fédération Dentaire Internationale) notation tooth numbering system [11].

**Table 1:** Teeth present in the oral cavity. FDI: Fédération Dentaire Internationale

FDI notation	Tooth name	Side
11	Permanent maxillary central incisor	Right
21	Permanent maxillary central incisor	Left
16	Permanent maxillary first molar	Right
26	Permanent maxillary first molar	Left
51	Deciduous maxillary central incisor	Right
61	Deciduous maxillary central incisor	Left
52	Deciduous maxillary lateral incisor	Right
62	Deciduous maxillary lateral incisor	Left
53	Deciduous maxillary canine	Right
63	Deciduous maxillary canine	Left
54	Deciduous maxillary first molar	Right
64	Deciduous maxillary first molar	Left
55	Deciduous maxillary second molar	Right
65	Deciduous maxillary second molar	Left
41	Permanent mandibular central incisor	Right
31	Permanent mandibular central incisor	Left
42	Permanent mandibular lateral incisor	Right
36	Permanent mandibular first molar	Left
46	Permanent mandibular first molar	Right
71	Deciduous mandibular central incisor	Left
81	Deciduous mandibular central incisor	Right
72	Deciduous mandibular lateral incisor	Left
82	Deciduous mandibular lateral incisor	Right
73	Deciduous mandibular canine	Left
83	Deciduous mandibular canine	Right
74	Deciduous mandibular first molar	Left
84	Deciduous mandibular first molar	Right
75	Deciduous mandibular second molar	Left
85	Deciduous mandibular second molar	Right

**Figure 3:**
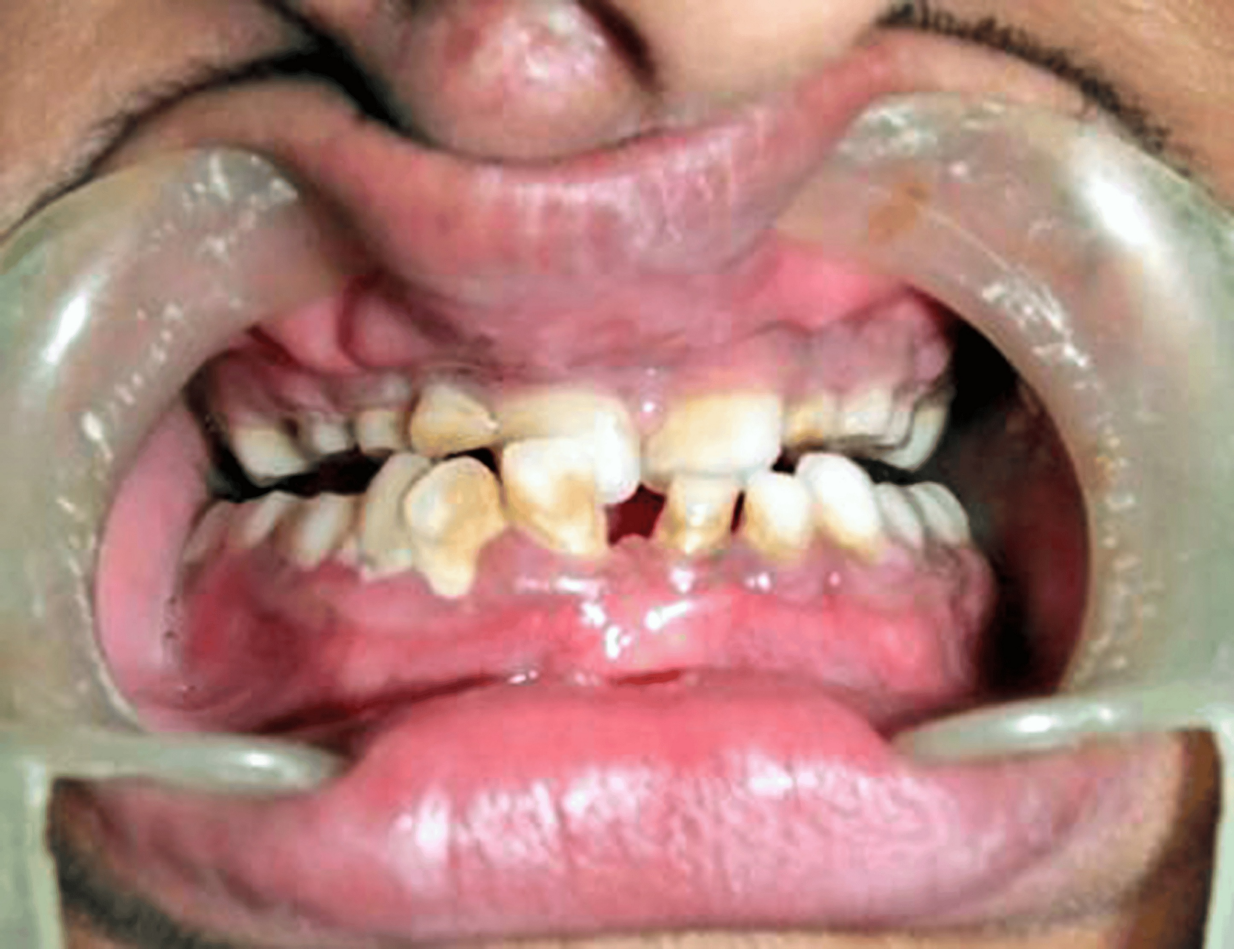
Showing over-retained deciduous teeth with hypoplastic enamel.

Occlusal caries with 26, grade II mobility with 71, 72, and grade III mobility with 52 were observed. Several over-retained deciduous teeth with hypoplastic enamel were present and poor oral hygiene with generalized gingivitis was also seen (Figure 3). Retrognathic maxilla and mandible, high arched palate, severe malocclusion with numerous missing and erupting teeth, and abnormal spacing are also seen.

**Figure 4:**
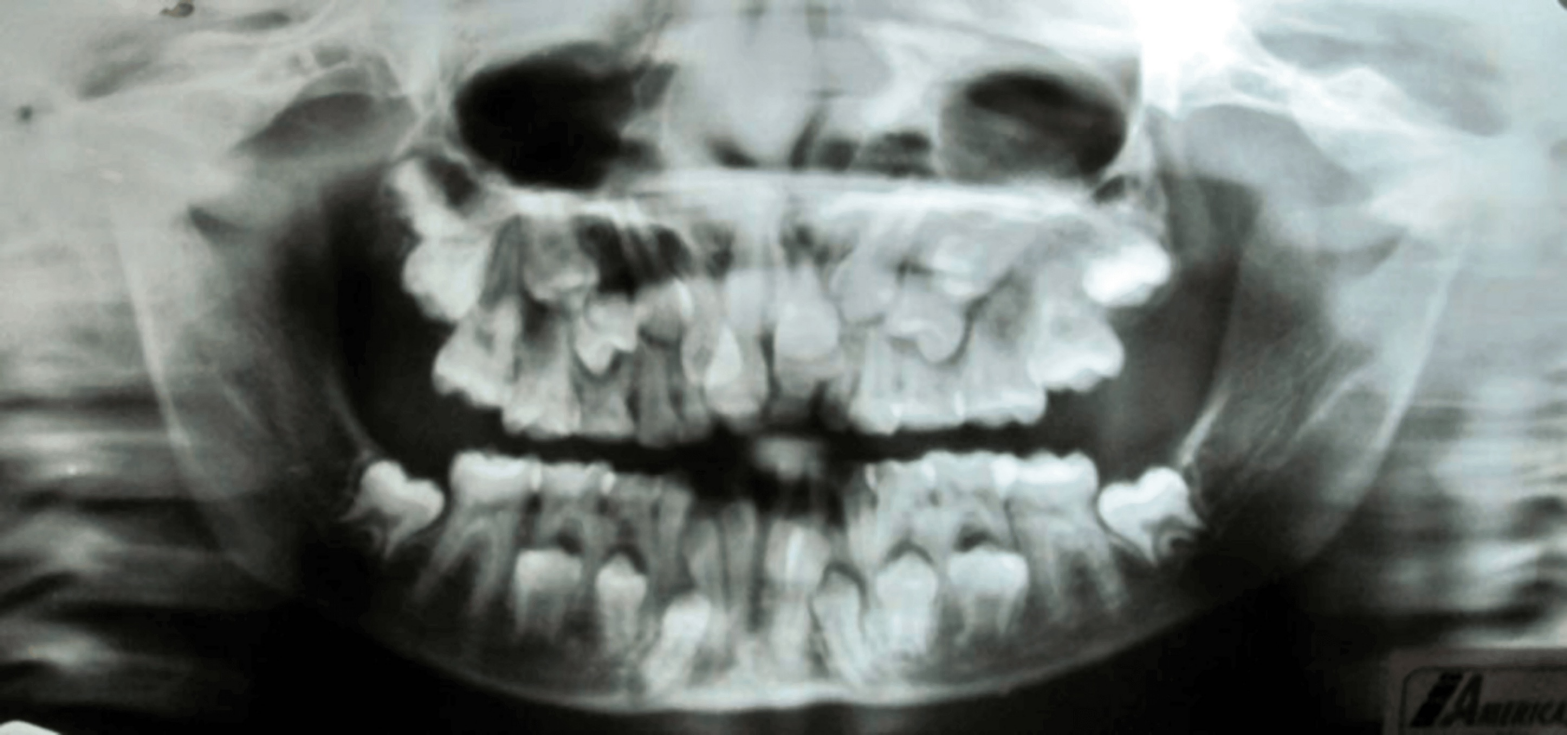
Orthopantomogram (OPG) showing multiple over-retained deciduous teeth, multiple unerupted supernumerary teeth, and impacted teeth.

On assessing the orthopantomogram (OPG), the classical signs of CCD were recognized. It shows coarse trabeculation with areas of increased density, slender pointed coronoid processes, a narrow ascending ramus, and impacted central incisors, lateral incisors, canines, and first and second premolars. The patient has around 42 teeth in both jaws. Some of the erupted teeth were over-retained deciduous teeth but most of them were unerupted and imitating a premolar. Maxillary sinuses were underdeveloped and gonial angles on the bilateral sides of the mandible were absent (Figure 4).

**Figure 5:**
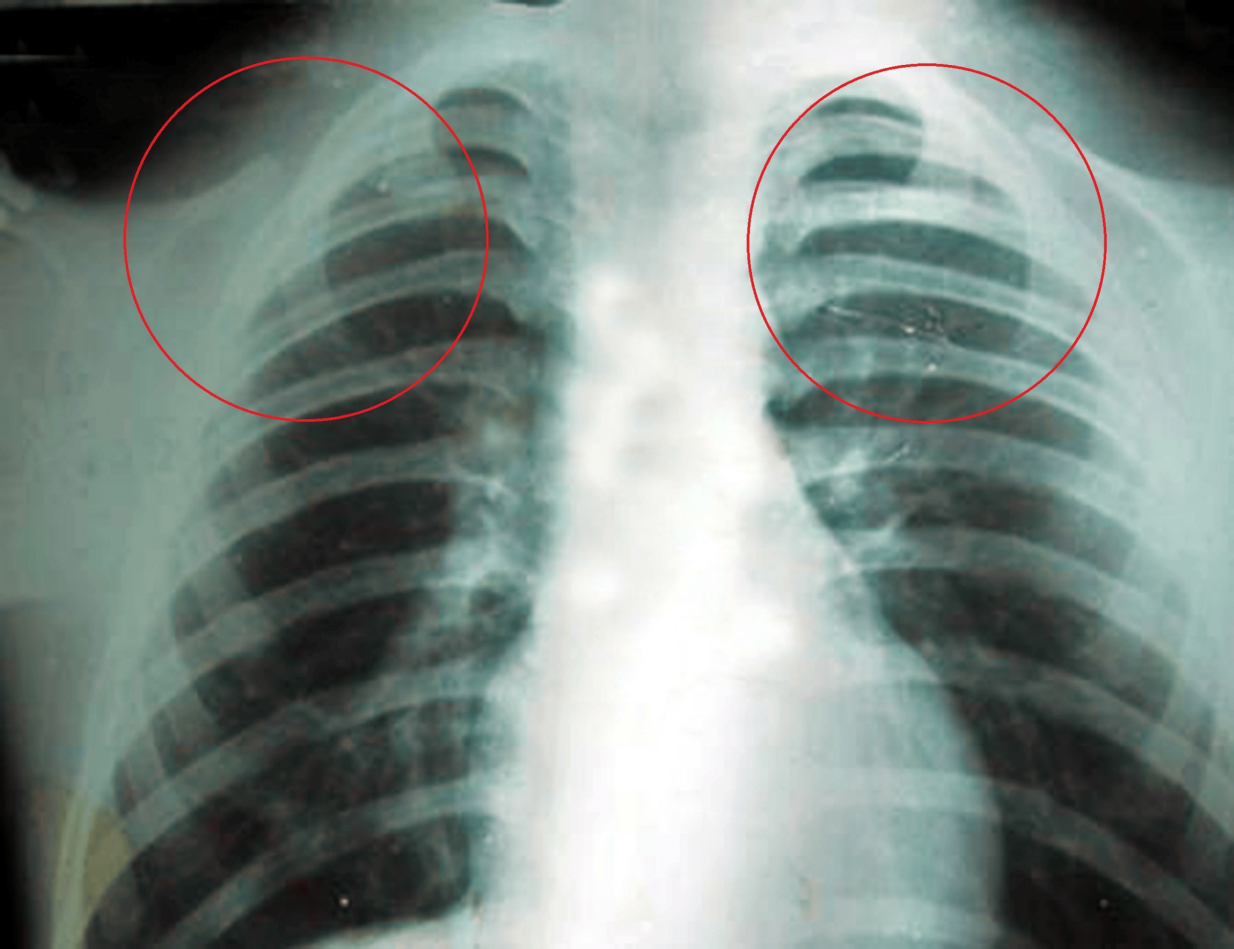
Posteroanterior (PA) view of chest x-ray showing complete absence of clavicles on both sides.

**Figure 6:**
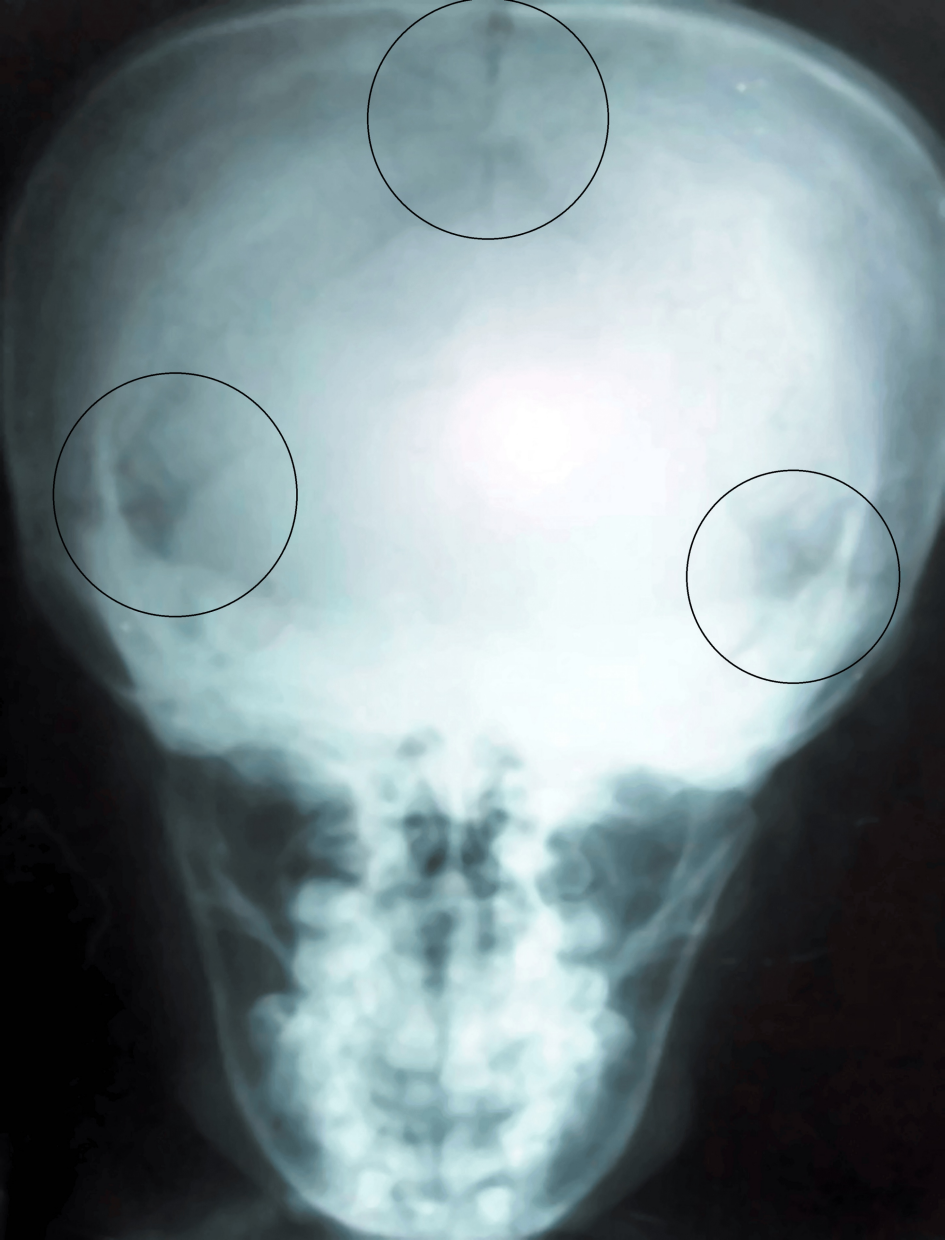
Posteroanterior (PA) view of skull showing metopic sutures, and the presence of multiple wormian bones.

The posteroanterior (PA) view of the chest radiograph confirms the bilateral absence of clavicles and narrow thorax (Figure 5). Apart from this, the PA view of the skull radiograph has shown the presence of multiple wormian bones and metopic sutures (Figure 6).

So based on clinical and radiological findings, the patient has been diagnosed with CCD. A complete dental treatment regime was explained and the patient never turned up for further treatment and follow-up. For the complete treatment of the patient, we included several specialists including orthodontists, oral surgeons, and prosthodontists. Firstly, the extraction of over-retained deciduous teeth and supernumerary teeth was planned. Then to induce the eruption of permanent teeth spacing was created for the eruption of permanent teeth and also surgical exposure of unerupted permanent teeth if needed was planned followed by orthodontic correction of teeth and esthetics of the patient. The case report is presented to report on the incidence, clinical and radiological manifestations, and various treatment considerations in treating a rare CCD case.

## Discussion

Patients with CCD can be diagnosed by clinical and radiological findings. Furthermore, with the identification of the mutation in the responsible gene, molecular genetic analysis is highly advised. It is also an important diagnostic tool for the early detection of CCD cases [8,12]. Studies have shown that in CCD patients 70% of them have shown point mutation associated with RUNX2 while 13% of patients show deletion. In 60%-70% of cases, mutations have been identified [9]. The main principal regulator that helps in the maturation and differentiation of chondrocytes and osteoblasts is RUNX2. The treatment plan for CCD patients majorly depends upon the patient's interests and demands, social and economic circumstances, the patient’s age, periodontal and endodontic health, and eruption stage of permanent dentition. Early diagnosis and timing of intervention are important in CCD patients. For the treatment of CCD patients, both physical and psychological aspects should be taken into consideration. The time of diagnosis of CCD may also be used as a criterion for selecting the right treatment regime to be followed [6].

Individuals diagnosed with CCD require long-term orthodontic and surgical treatments, which increases the difficulty in dental management. So, a comprehensive treatment approach is considered involving maxillofacial surgery, prosthodontics, and orthodontics. For patients with CCD, recent advances in dentistry have improved treatment options, prognosis, and results. Dental implants, for example, are the best and most effective option for replacements of teeth that cannot be orthodontically guided into the arch as well as minimally invasive orthodontic tooth movement. According to the study conducted by Ahmad et al., full mouth rehabilitation was done using basal implants in CCD patients thus improving the quality of life [13].

Orthognathic surgery also corrects CCD skeletal deformity, though it is expensive, the treatment outcomes are achieved in the shortest time. Prosthetic treatment is another form of therapy option for CCD patients. Atil et al. described a case on the use of implant-supported fixed dental prosthesis for oral reconstructions in middle-aged CCD patients [14]. Prosthetic therapy can restore oral esthetics and functions in a shorter period and also eliminates various problems caused by orthodontic and surgical treatments but this treatment is only suitable for elderly patients.

If the bone density is below normal then one should consider the treatment with vitamin D and calcium tablets. Screening for osteoporosis should be conducted at an early age, as the bone density mass is usually at its peak during the early decades of life. Therefore, early identification of CCD will prove greatly advantageous in ensuring early therapy and will have a larger role to play in enabling a more successful craniofacial reconstruction and functions [15]. The mutation is more likely to be inherited by the offspring of CCD patients. Therefore, genetic counseling is important for young adults who have been affected. Therefore, it is also important to do proper counseling of the patients and their families.

## Conclusions

Early diagnosis of cleidocranial dysplasia (CCD) is important for effective management. Molecular gene testing plays an important role in confirming the diagnosis and in further counseling the families with the disease. Multidisciplinary management involving dental specialists, orthopedic surgeons, and geneticists is crucial in treating CCD. Frequent follow-up and individual treatment care plans play a crucial role in monitoring and managing potential complications like dental issues, etc. Therefore, in conclusion through organized and systemic care and early intervention, patients with CCD can have better functional outcomes and further improve their quality of life.
